# The increment of micronucleus frequency in cervical carcinoma during irradiation in vivo and its prognostic value for tumour radiocurability

**DOI:** 10.1038/sj.bjc.6690569

**Published:** 1999-07

**Authors:** M Widel, S Jędruś, S Owczarek, M Konopacka, B Lubecka, Z Kołosza

**Affiliations:** Department of Experimental and Clinical Radiobiology, Clinic of Oncological Gynaecology and Department of Epidemiology, Centre of Oncology, Maria Skłodowska-Curie Institute Gliwice, 15 Armii Krajowej Str., 44-101 Gliwice, Poland

**Keywords:** cervical carcinoma, radiotherapy, micronucleus assay, predictive value

## Abstract

A potential usefulness of micronucleus assay for prediction of tumour radiosensitivity has been tested in 64 patients with advanced stage (II B–IV B) cervical carcinoma treated by radiotherapy. The study of cellular radiosensitivity in vitro was conducted in parallel with the study of cellular damage after tumour irradiation in vivo. Radiosensitivity of in vitro cultured primary cells isolated from tumour biopsies taken before radiotherapy was evaluated using cytokinesis-block micronucleus assay. Frequency of micronuclei per binucleated cell (MN/BNC) at 2 Gy was used as a measure of radiosensitivity. Radiation sensitivity in vivo was expressed as per cent increment of micronucleus frequency in cells isolated from biopsy taken after 20 Gy (external irradiation, 10 × 2 Gy) over the pre-treatment spontaneous micronucleus level and was called MN20. Very low correlation (*r* = 0.324) was observed between micronucleus frequency in vitro and in vivo. Although micronucleus frequency at 2 Gy differed widely between tumours evaluated (mean MN/BNC was 0.224; range 0.08–0.416), no significant correlation was observed between this parameter and clinical outcome. The average increment of micronucleus frequency after 20 Gy amounted to 193% of spontaneous level (range 60–610%) and was independent of spontaneous micronucleation before radiotherapy. In contrast to in vitro results, these from in vivo assay seem to have a predictive value for radiotherapy of cervix cancer. The micronucleus increment in vivo that reached at least 117.5% of pretreatment value (first quartile for MN20 data set) correlated significantly with better tumour local control (*P* < 0.008) and overall survival (*P* < 0.045). Our results suggest that evaluation of increment of micronucleus frequency during radiotherapy (after fixed tested dose of 20 Gy) offers a potentially valuable approach to predicting individual radioresponsiveness and may be helpful for individualization of treatment strategy in advanced stage cervical cancer. © 1999 Cancer Research Campaign


*British Journal of Cancer* (1999) **80**(10), 1599–1607
© 1999 Cancer Research Campaign
Article no. bjoc.1999.0569

### The increment of micronucleus frequency in cervical carcinoma during irradiation in vivo and its prognostic value for tumour radiocurability

**M Widel[1], S Jędruś[2], S Owczarek[2], M Konopacka[1], B Lubecka[1] and Z Kołosza[3]**

[1]Department of Experimental and Clinical Radiobiology, [2]Clinic of Oncological Gynaecology and [3]Department of Epidemiology, Centre of Oncology, Maria Skłodowska-Curie Institute Gliwice, 15 Armii Krajowej Str., 44-101 Gliwice, Poland

**Summary** A potential usefulness of micronucleus assay for prediction of tumour radiosensitivity has been tested in 64 patients with advanced stage (II B–IV B) cervical carcinoma treated by radiotherapy. The study of cellular radiosensitivity in vitro was conducted in parallel with the study of cellular damage after tumour irradiation in vivo. Radiosensitivity of in vitro cultured primary cells isolated from tumour biopsies taken before radiotherapy was evaluated using cytokinesis-block micronucleus assay. Frequency of micronuclei per binucleated cell (MN/BNC) at 2 Gy was used as a measure of radiosensitivity. Radiation sensitivity in vivo was expressed as per cent increment of micronucleus frequency in cells isolated from biopsy taken after 20 Gy (external irradiation, 10 × 2 Gy) over the pre-treatment spontaneous micronucleus level and was called MN20. Very low correlation ($r = 0.324$) was observed between micronucleus frequency in vitro and in vivo. Although micronucleus frequency at 2 Gy differed widely between tumours evaluated (mean MN/BNC was 0.224; range 0.08–0.416), no significant correlation was observed between this parameter and clinical outcome. The average increment of micronucleus frequency after 20 Gy amounted to 193% of spontaneous level (range 60–610%) and was independent of spontaneous micronucleation before radiotherapy. In contrast to in vitro results, these from in vivo assay seem to have a predictive value for radiotherapy of cervix cancer. The micronucleus increment in vivo that reached at least 117.5% of pretreatment value (first quartile for MN20 data set) correlated significantly with better tumour local control ($P < 0.008$) and overall survival ($P < 0.045$). Our results suggest that evaluation of increment of micronucleus frequency during radiotherapy (after fixed tested dose of 20 Gy) offers a potentially valuable approach to predicting individual radioresponsiveness and may be helpful for individualization of treatment strategy in advanced stage cervical cancer.

**Keywords:** cervical carcinoma; radiotherapy; micronucleus assay; predictive value

Radiocurability of human tumours depends on many tumour- and host-related factors. Among tumour-related factors, intrinsic radiosensitivity is believed to be one of the most important and relatively independent determinants of tumour response to radiotherapy (Fertil and Malaise, 1985). It has been shown, especially for cervical carcinomas, that intrinsic radiosensitivity measured as surviving fraction of clonogens at 2 Gy (SF2) strongly correlated with clinical outcome, and both local control and patient survival (West et al, 1992, 1993, 1997). Also initial slopes of survival curves (α values) for cervix carcinoma and head and neck cancer were significantly correlated with local control and overall survival (Girinsky et al, 1992). A similar trend, although insignificant, was also observed by the same authors for SF2.

Radiotherapy is a method of choice in advanced stages of cervix cancer, also at our Centre. However, local recurrences are often the reason of treatment failure. Since a great inter-tumour heterogeneity in radiosensitivity has been observed even within one histological type of cervical tumour, e.g. SF2 = 0.47 (coefficient of variation, cv = 38%) for squamous cell carcinoma and SF2 = 0.59 (cv = 46%) for adenocarcinoma (Davidson et al, 1990), one can expect that this heterogeneity contributes to difference in clinical response. Therefore, the ability to select patients with a high probability of recurrence on the basis of cellular radiosensitivity would be useful in deciding about adjuvant treatment.

The clonogenic or population growth assays are most frequently used for evaluation of cellular radiosensitivity. They are, however, rather laborious and long-lasting methods, not suitable for routine application. Therefore, some quicker alternative clinically useful methods are required. Different non-clonogenic assays of DNA damages are currently tested as alternatives. Among them, becoming popular and promising are methods measuring DNA double-strand breaks induction and rejoining (Schwartz et al, 1991, 1996; Whitaker et al, 1995), as well as cytogenetic assay of micronuclei (MN), (Shibamoto et al, 1991, 1994, 1998; Bakker et al, 1993; Zölzer et al, 1995). The micronucleus assay elicited much interest due to its relative simplicity and rapidity in comparison with clonogenic survival assay. Since micronuclei arise from disturbed genetic materials such as acentric chromosomal fragments (Heddle and Carrano, 1977), or even whole chromosomes (Weissenborn and Streffer, 1991), the cells containing micronuclei are potentially dead. Some experiments on established normal cell lines (Midander and Revesz, 1980), on human tumour xenografts (Shibamoto et al, 1991) and on early passages of human tumour cells (Wandl et al, 1989) indicated good correlation between radiation-induced micronucleus frequency and clonogenic cell survival. Our own study on

*Received 11 August 1998*
*Revised 5 January 1999*
*Accepted 11 February 1999*

*Correspondence to:* M Widel malignant melanomas also indicated good correlation between both end-points (Widel et al, in preparation). However, results of studies by Bush and McMillan (1993), Villa et al (1994) and Champion et al (1995) have shown some divergence between micronucleus frequency and clonogenic cell survival. On the other hand, Shibamoto et al (1994, 1998) found a positive correlation between frequency of micronuclei induced in vitro in different types of human tumours and an early clinical response to radiotherapy. Furthermore, it has been shown by conventional micronucleus assay that an increase of micronucleus frequency in head and neck cancer during radiotherapy is related to better prognosis, in comparison with patients whose tumours indicated no change in micronuclei (Zamboglou et al, 1992). Similarly in cervix cancer, increase of MN frequency during radiotherapy correlated, although insignificantly, with better prognosis (Zölzer et al, 1995). However, increase of MN frequency together with diminution of S phase fraction during radiotherapy was significantly correlated with higher 5-year survival (Zölzer et al, 1995). Thus, definite elucidation of predictive value of micronucleus assay needs further experimentation.

In our study we attempted to compare sensitivity of advanced stage cervical carcinomas to induction of micronuclei after irradiation in vitro (2–4 Gy) and in vivo (10 × 2 Gy) and to analyse correlation between these biological parameters and clinical outcome after radiotherapy. The results from 64 patients with a mean 2.5-year follow-up are presented.

### MATERIALS AND METHODS

### Patients and treatment

Sixty-four patients with stage II B–IV (FIGO) cervical tumours were selected for study between January 1992 and November 1995. Thirteen patients had stage II B tumours, 49 had stage III B and two had stage IV B with para-aortic lymph node involvement. All tumours were squamous cell carcinomas. The mean age of patients was 47.8 with a range 33–71 years. Mean haemoglobin (Hb) level prior to radiotherapy was 12.9 g $dl^{-1}$ (8 M $l^{-1}$) with a range 8.4–16.5 g $dl^{-1}$ and patients were transfused if the Hb level was lower than 10 g $dl^{-1}$ before radiotherapy, or if it dropped below this level during the treatment.

All patients were treated by radiotherapy. According to the scheme accepted in our Centre, the treatment starts from external beam irradiation (Co-60 γ-rays, or 9 or 21 MV fotons) to the whole pelvis without shielding using 2 Gy per fraction (2 × 1 Gy) given through opposed anterior-posterior fields. After 30 Gy a central shielding was used to protect bladder and rectum. External irradiation up to 48–52 Gy (increasing dose in more advanced disease) was followed by intracavitary HDR-afterloading brachytherapy with Ir-192. Eight to nine fraction of 5 Gy calculated to the Point A were given at 3-day intervals. Total dose from brachytherapy was 40–45 Gy to point A.

Tumour biopsies were taken before starting the radiotherapy and after 20 Gy (10 fraction of 2 Gy from external irradiation). The follow-up schedule applied in our Centre was 1 month after finishing the treatment and every 2 months during the first year, followed by 3-monthly controls in the second year and 6-monthly controls in subsequent years. The study was approved by local Medical Ethical Committee and all patients gave informed consent before entry.

### Tumour processing and micronucleus assay in vitro

Tumour biopsies for cytokinesis-block micronucleus assay (CBMN-assay) in vitro were obtained a few hours before beginning the radiotherapy. The methods of tumour processing in order to obtain single-cell suspension for cell culture were presented elsewhere (Widel et al, 1997). Briefly, tumour specimens (50–200 mg) were washed twice in antibiotic-rich medium and minced with scissors. A small part of minced tissue (10–20 mg) was used for assessment of spontaneous micronucleation before radiotherapy (see the next paragraph). The rest was digested in collagenase/dispase (Boehringer-Mannheim, Germany; 0.5 mg $ml^{-1}$ in physiological saline) for 30 min at 37°C. Cells were filtered through a stainless mesh (50 μm), spun down and re-suspended in growth medium (GM). The GM was Dulbecco's modified medium and Ham's nutrient mixture F12 (Sigma, Germany) enriched with 20% of fetal bovine serum (FBS; Gibco, UK), hormones (5 μg $ml^{-1}$ insulin, 5 μg $ml^{-1}$ hydrocortisone, both from Sigma) and 5 ng $ml^{-1}$ epidermal growth factor, 10 μg $ml^{-1}$ transferrin and 1 μg $ml^{-1}$ fibronectin (all from Boehringer-Mannheim, Germany). Three millilitres of cell suspension at concentration 1–2 × $10^5$ viable cells per 1 ml (viability examined by trypan blue exclusion) were plated per dish in a series of nine dishes (Nunc, 25 $cm^2$) and incubated for attachment and spreading. Usually after 24 h incubation, well-attached and spread cells were irradiated with 2 and 4 Gy (Co-60 γ-rays, dose rate 1 Gy $min^{-1}$, room temperature). Non-irradiated control dishes were sham exposed at the same time to keep changes in pH and temperature similar. Immediately after irradiation medium was removed and 5 ml of fresh GM containing cytochalasin B (2 μg $ml^{-1}$; Sigma, USA) was added. Incubation was terminated at 96 h after irradiation and cells were fixed in situ with glutaraldehyde and stained with Schiffs' reagent, as presented elsewhere (Shibamoto et al, 1991; Widel et al, 1997). Micronuclei were scored in binucleated cells under Zeiss-Opton microscope (1000 × magnification) using bright field or/and phase contrast equipment. Tumour cells were differentiated with normal fibroblasts on the basis of squamocellular appearance and morphological features characteristic for tumour cells, such as: high ratio of nucleus/cytoplasm volume, irregularity in nucleus shape and in density of staining (Shibamoto et al, 1994, 1998). At least 100 binucleated cells (BNC) were scored per dish. Since binucleation frequency was rather low in this tumour type, it was necessary to score a total number of 500–1000 cells; however, in few cases it was not even possible to find 100 BNC per 1000 cells. Three dishes were used per treatment dose. The BNC with one or more micronuclei were counted and frequency of binucleated cells with micronuclei (% BNC with MN) as well as number of micronuclei per binucleated cell (MN/BNC) were calculated.

### Micronucleus assay in vivo

To evaluate the incidence of micronuclei after irradiation in vivo, the tested dose of 20 Gy given to the tumour (10 daily fractions of 2 Gy from external source) was chosen. For this assay biopsies were taken two times, first before radiotherapy (the same biopsy as for MN assay in vitro) and again after 20 Gy. Second biopsies were taken approximately after 24 (±1) h after 10th fraction of 2 Gy. In both cases tumour tissue was minced with scissors and pressed with tweezers through a nylon 50-μm mesh while rinsing with phosphate-buffered saline. Cells were spun down,

*© 1999 Cancer Research Campaign*

fixed in ice-cold 96% ethanol and kept in refrigerator. Before MN-analysis, cells were refixed in acetic acid:methanol 1:3 (v/v) and microscopic slides were prepared. After being air-dried overnight, slides were stained according to standard Pappenheim procedure with May–Grünwald Giemsa stain (POCH, Gliwice, Poland). This method of staining was preferred to fluorescent dyes used by others in somewhat similar studies (Zölzer et al, 1995) since it allows differentiation between tumour and non-tumour cells (infiltrating lymphocytes, granulocytes and macrophages). Micronuclei were scored at least in 1000 tumour cells and expressed as number of micronuclei per cell. Spontaneous level of micronuclei before radiotherapy (MNSP) was considered as 100% and level of micronuclei after 20 Gy (MN20) was considered as relative increment for particular tumour and was calculated according to the equation:

$$\text{MN20 (\%)} = \frac{\text{MN/cell after 20 Gy}}{\text{MN/cell before RT}} \times 100$$

### Statistical analysis

Local control was evaluated from the date of finishing the brachytherapy to the date of appearance of recurrence. Survival was measured from the date of finishing the brachytherapy to the date of last follow-up or death. Estimates of cumulative proportion of recurrences and overall survival were done by the Kaplan–Meier method using Statistica v.5.0 software. The differences between particular arms presented in the Figures were analysed for significance using the log-rank test. The differences between data sets for patients who died and those who were alive at the time of analysis were analysed using Student's *t*-test for significance (Table 1).

### RESULTS

Six patients of 64 selected for the study did not complete radiotherapy because of tumour progression and/or complications caused by advanced disease. Data from all 58 patients who completed full course of radiotherapy were included in the Kaplan–Meier analysis of survival and local control probability. Two of them with locally controlled tumours and in good general status were lost after 19 and 22 months of observation. The rest had a minimum 2.5-year post-treatment follow-up (range 28–66 months). All patients were included in analysis of age, haemoglobin level and some biological data presented in Table 1. The frequency of micronuclei was evaluated after irradiation of tumour in primary culture (2 and 4 Gy) and in vivo using 20 Gy as a tested dose. It was possible to evaluate micronucleus frequency after 2 Gy in vitro in 43/64 tumours (67%), where cell cultures were successful. Because of insufficient amount of cells isolated from biopsies, data at 4 Gy were obtained for 35 tumours only. The main data obtained from in vitro assay are summarized in Table 2. We observed great variation in binucleation yield in non-irradiated control cells as well as in irradiated ones. The mean percentage of BNC at 0 Gy was 15.8% (range 7.0–45.0) indicating that dividing fraction in this tumour type is rather low. Despite a long time of incubation with cytochalasin B (96 h chosen in preliminary study as optimal), the yield of multinucleated cells was negligible (0–3%, data not presented). The average yield of BNC dropped to 12.52 and 10.91% at 2 Gy and 4 Gy, respectively, due to inhibition of cell cycle progression. The micronucleus frequency expressed as either % BNC with MN or MN/BNC increased with increasing dose (Table 2). The very weak correlation was found between % BNC and frequency of BNC with MN at 2 Gy ($r = 0.169$) and 4 Gy ($r = 0.219$). Corresponding correlation coefficients for % BNC vs MN/BNC were 0.367 at 2 Gy and 0.220 at 4 Gy. Since data at

**Table 1** Comparison of some biological and clinical data between the groups of patients who died and those who are alive at the time of analysis

| Parameter evaluated | Group of patients | | Significance[a] |
|---|---|---|---|
| | Dead | Alive | |
| Total number of patients | 29 | 35 | |
| FIGO stage: II B | 1 | 12 | |
| III B | 28 | 21 | |
| IV B | 0 | 2 | |
| Age of patients[b] | 48.2 ± 9.5 | 47.4 ± 8.4 | $P = 0.7$ |
| Haemoglobin level at the start of treatment (g/dl)[b] | 12.6 ± 1.9 | 13.4 ± 1.5 | $P = 0.1$ |
| % BNC at 0 Gy[b] | 17.5 ± 10.25 ($n = 20$) | 14.39 ± 3.39 ($n = 23$) | $P > 0.2$ |
| % BNC with MN at 2 Gy[b,c] | 17.76 ± 5.43 ($n = 20$) | 16.42 ± 5.16 ($n = 23$) | $P = 0.4$ |
| MN/BNC at 2 Gy in vitro[b,c] | 0.234 ± 0.07 ($n = 20$) | 0.216 ± 0.06 ($n = 23$) | $P = 0.4$ |
| Spontaneous micronucleus frequency before radiotherapy (MNSP)[b] | 3.28 ± 1.3 ($\times 10^{-2}$) ($n = 29$) | 2.74 ± 1.2 ($\times 10^{-2}$) ($n = 35$) | $P = 0.1$ |
| Percent increment of micronucleus frequency after 20 Gy in vivo in relation to MNSP (MN20)[b] | 174.04 ± 83.93 ($n = 27$) | 207.1 ± 110.22 ($n = 34$) | $P = 0.2$ |
| Survival time[b] after radiotherapy (months) | 15.89 ± 10.20 | 42.45 ± 11.42 | $P < 0.001$ |

[a]Student's *t*-test; [b]mean ± s.d.; [c]corrected for 0 Gy data.

© 1999 Cancer Research Campaign

**Table 2** The main results of cytokinesis-block micronucleus assay in primary culture of cervical carcinomas

| Parameter | | Dose (Gy) | | | | |
|---|---|---|---|---|---|---|
| | | 0 | 2 | 4 | 2[a] | 4[a] |
| % BNC | Mean | 15.84 | 12.52 | 10.91 | | |
| | ± s.d. | 8.50 | 5.10 | 4.44 | | |
| | Range | 7.00–45.00 | 6.0–33.00 | 6.00–28.00 | | |
| | cv (%) | 54 | 41 | 41 | | |
| | *n* | 43 | 43 | 34 | | |
| % BNC with MN | Mean | 8.50 | 25.35 | 32.90 | 17.04 | 24.20 |
| | ± s.d. | 4.90 | 6.08 | 10.42 | 5.27 | 8.53 |
| | Range | 0.00–17.00 | 13.00–41.00 | 14.00–66.00 | 9.00–30.00 | 10.00–50.00 |
| | cv (%) | 57 | 24 | 32 | 31 | 35 |
| | *n* | 43 | 43 | 34 | 43 | 34 |
| MN/BNC | Mean | 0.096 | 0.315 | 0.415 | 0.224 | 0.321 |
| | ± s.d. | 0.062 | 0.090 | 0.132 | 0.066 | 0.116 |
| | Range | 0.00–0.257 | 0.160–0.626 | 0.180–0.683 | 0.080–0.416 | 0.109–0.584 |
| | cv (%) | 65 | 29 | 32 | 30 | 36 |
| | *n* | 43 | 43 | 34 | 43 | 34 |

s.d., standard deviation; cv, coefficient of variation, [a]after correction for 0 Gy data.

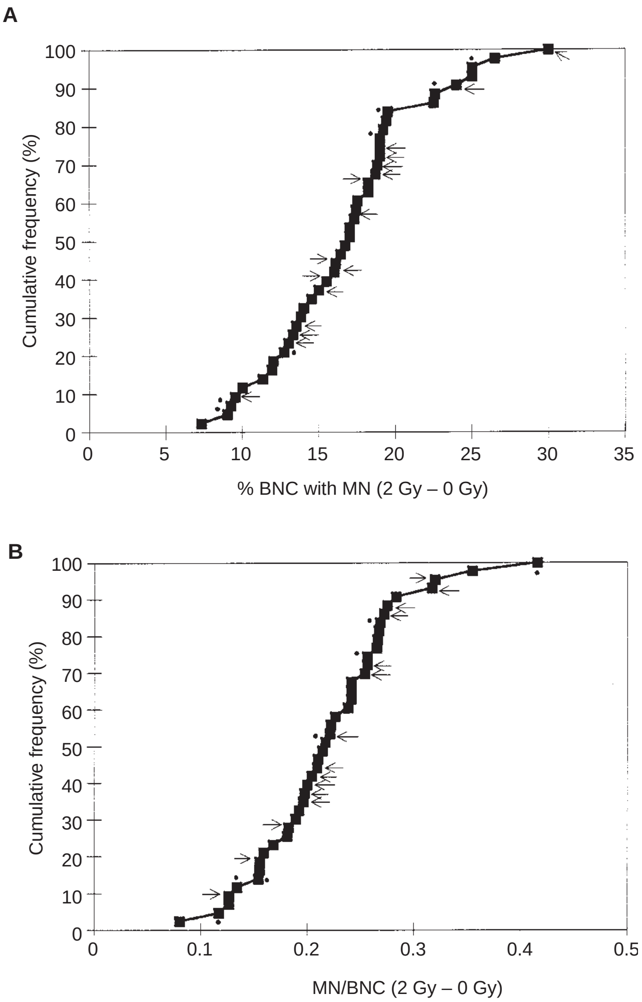

**Figure 1** Cumulative frequency distribution of the percentage of binucleated cells with micronuclei (**A**) and number of micronuclei per binucleated cells (**B**) estimated in vitro for 43 advanced stage cervical carcinomas. Local recurrences and distant metastases are indicated by arrows and dots respectively

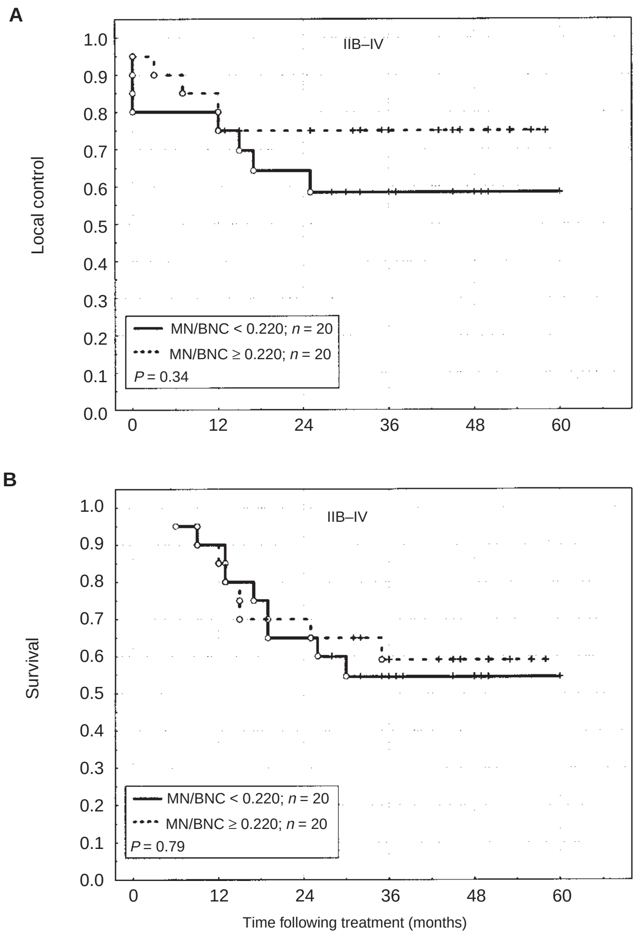

**Figure 2** Local control (**A**) and overall survival (**B**) probability vs. micronucleus frequency at 2 Gy (after correction for 0 Gy) estimated in vitro. The data for patients who completed full course of radiotherapy are divided into two groups for those below and above or equal to the median value of MN/BNC

© 1999 Cancer Research Campaign

**Table 3** Correlation between some data of micronucleus assay in vitro and in vivo

| Correlation between parameters | Correlation coefficient (*r*) | Number of tumours/ evaluated |
|---|---|---|
| % BNC (2 Gy) and % BNC with MN (2 Gy)[a] | 0.169 | 43 |
| % BNC (2 Gy) and MN/BNC (2 Gy)[a] | 0.367 | 43 |
| % BNC with MN (2 Gy)* and MN/BNC (2 Gy)[a] | 0.710 | 43 |
| % BNC (4 Gy) and % BNC with MN (4 Gy)[a] | 0.219 | 34 |
| % BNC (4 Gy) and MN/BNC (4 Gy)[a] | 0.220 | 34 |
| MN/BNC (0 Gy) and MN/BNC (2 Gy) | 0.660 | 43 |
| MN/BNC (0 Gy) and MNSP | 0.176 | 43 |
| MN/BNC (2 Gy)[a] and MN20 | 0.324 | 41 |
| MNSP and MN20 | 0.290 | 58 |

[a] Corrected for 0 Gy data.

4 Gy were obtained for 34 tumours only (53%) they were not further analysed and considered as not representative for the whole group. Figure 1 shows the cumulative frequency distribution of % BNC with MN and MN/BNC at 2 Gy. Both plots indicate very similar shape and normal distribution of values. The local recurrences and metastases that are indicated by arrows and dots respectively, are almost equally distributed below and over the median values in each plot. However, these indicators of metastases and recurrences are not ideally placed in the same spots of both plots since there is no ideal correlation between % BNC with MN and MN/BNC ($r$ = 0.710, Table 3). The MN/BNC at 2 Gy was further used for analysis of correlation between clinical outcome and radiation sensitivity of tumour cells. Figure 2 presents the relationships between tumour local control or patient overall survival and MN/BNC at 2 Gy corrected for 0 Gy. The results from patients who completed full course of radiotherapy were divided according to the median (equal 0.220). Although tumour local control probability at 58th month was almost 20% higher for the group with MN/BNC over the median, significance has not been achieved (Figure 2A). Furthermore, there were no differences in overall survival between both arms (Figure 2B). If we split the data into four quartiles, we could not find any correlation with clinical outcome either (data not presented). Also average binucleation frequency which indirectly reflects proliferative activity of tumours did not influence clinical results and was only slightly higher in the group of patients who died than in the group of patients who were alive at the time of analysis (Table 1). On the basis of data obtained for 40 patients who completed the full course of radiotherapy we can state that micronucleus assay in vitro will have limited usefulness for predicting treatment outcome at least for patients with cervical carcinomas.

The micronucleus frequency after 20 Gy in vivo (MN20) was estimated for 61 tumours and was expressed as the per cent increment in relation to spontaneous micronucleus frequency (MNSP). Spontaneous micronucleus frequency was estimated in all 64 tumours. Median value for MNSP was $2.8 \times 10^{-2}$ per cell (range $0.16–6.8 \times 10^{-2}$ per cell). We observed that better prognosis was connected with the moderate values of MNSP, as presented in Figure 3, where local control and overall survival were analysed in relation to MNSP stratified according to four quartiles. It can be seen that patients with either extremely high or low values of MNSP mostly belonged to the group with shorter time of loco-regional control (Figure 3A). Significant difference in local control

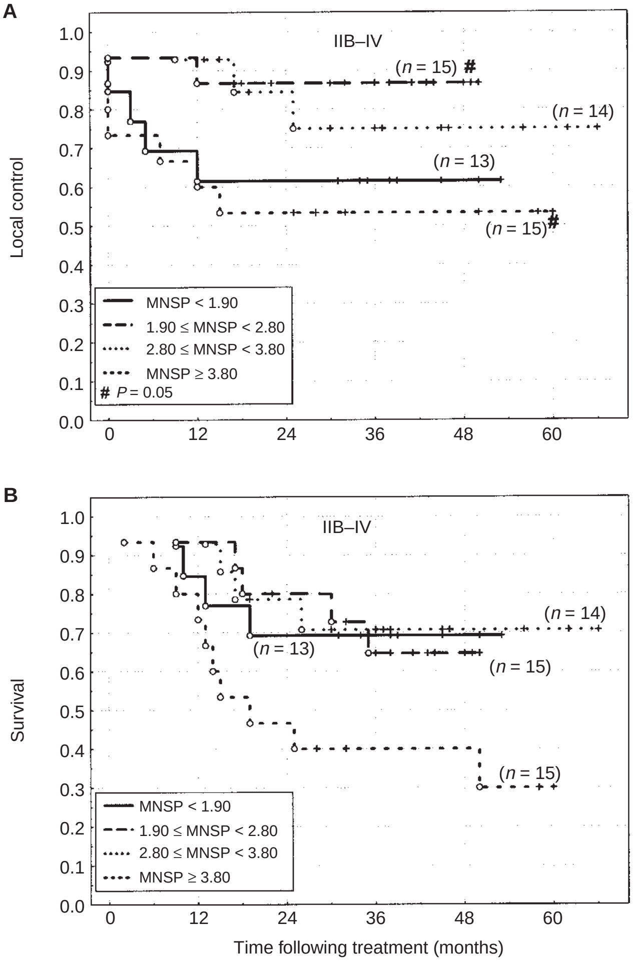

**Figure 3** Local control (**A**) and overall survival (**B**) probability vs. frequency of spontaneous micronuclei per cell. The data for patients who completed full course of radiotherapy are stratified according to the four quartiles of MNSP (the values of micronuclei per cell given in the frame should be multiplied by $10^{-2}$). Significant differences were obtained only for two arms signed by # in **A**; the differences between others were not significant probability ($P$ = 0.049) was achieved only between arms representing moderate value ($1.9 \leq$ MNSP $<2.8 \times 10^{-2}$ per cell) and arms representing MNSP $\geq 3.8 \times 10^{-2}$ per cell (Figure 3A). Survival of patients was also considerably lower for the group with MNSP above the fourth quartile (Figure 3B). However, precise analysis of the data for tumours represented by the lowest arm in Figure 3 has revealed that nine of 15 tumours with MNSP above the fourth quartile (MNSP $> 3.8 \times 10^{-2}$ per cell) simultaneously showed the low increase of micronucleus number after 20 Gy, lower than median value for this parameter set, and six of these tumours had MN20 below first quartile value. Nevertheless, very low correlation was observed in general between MNSP and MN20 ($r$ = 0.290) for 58 tumours (Table 3). Data presented in Figures 4–6 indicate that increment of micronucleus frequency in vivo after 20 Gy dose (MN20) may have predictive value for treatment outcome. Figure 4 presents the local control (Figure 4A) and overall survival probability (Figure 4B) in relation to MN20 stratified according to the median value (163.5%). Data combine all stage patients who completed full course of radiotherapy. A significantly higher ($P$ = 0.009), at least 3-year, local control was

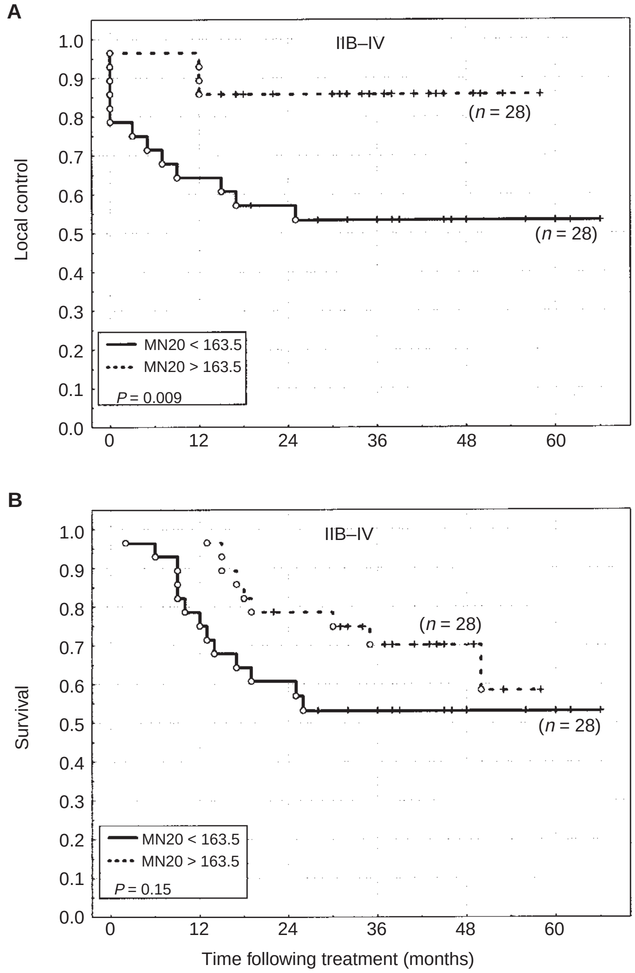

**Figure 4** Local control (**A**) and overall survival (**B**) probability vs increment of micronucleus frequency after 20 Gy in vivo in patients with stage II B–IV cervical carcinoma who completed full course of radiotherapy. The data are stratified according to the median value for MN20 equal to 163.5% of pretreatment spontaneous micronucleus frequency

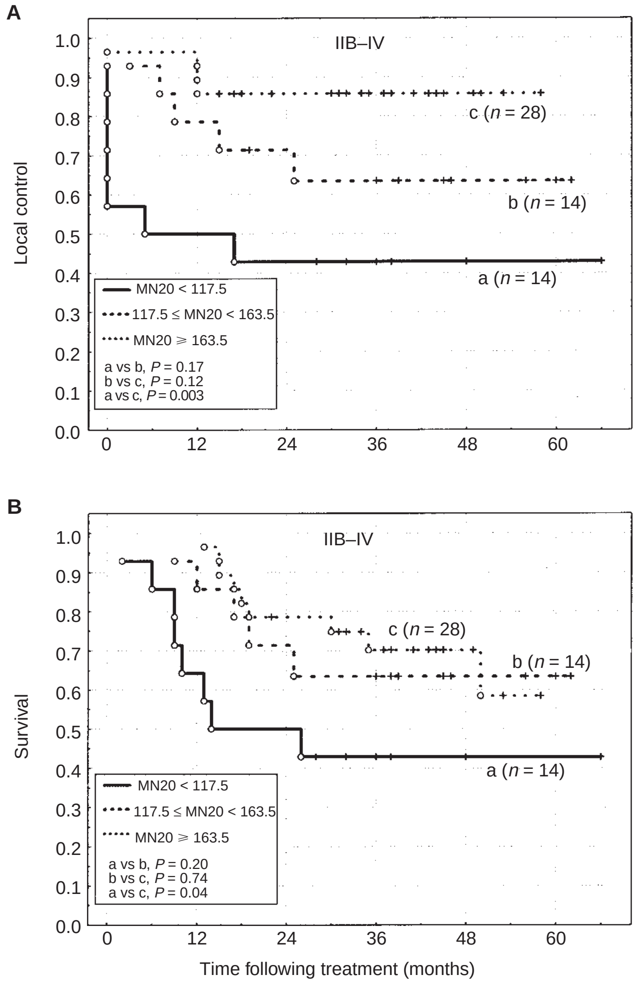

**Figure 5** Local control (**A**) and overall survival (**B**) probability vs increment of micronucleus frequency after 20 Gy in vivo for stage II B–IV tumours. The upper arm represents tumours with MN20 above the median; middle arm represents MN20 between the median and first quartile and lower arm represents MN20 below the first quartile achieved for the group of patients whose tumours responded by higher increment of micronucleus frequency (MN20 above the median, upper arm in Figure 4A) than for the group whose tumours showed MN20 below the median (lower arm in Figure 4A). Only one tumour in superior arm progressed despite the radiation treatment and three others recurred within the first year after radiotherapy. In the group of patients with MN20 lower than the median, six tumours progressed or stagnated in spite of radiation treatment and seven others recurred within 2 years (lower arm in Figure 4A). The significance was not achieved for overall survival (Figure 4B) probably because of generally shorter follow-up time for patients who belonged to the group with MN20 over the median. The similar trend was also observed for a narrower group of patients with stage III–IV of disease which included 42 patients in stage III B and two in stage IV (data not shown). Significance was obtained for local control ($P = 0.019$) but not for overall survival probability ($P = 0.29$).

Figure 5 clearly indicates that higher local control can be achieved when tumours respond to irradiation by high increase of micronucleation during radiotherapy (MN20 ≥ 163.5%, arm c, Figure 5A). High significant difference ($P = 0.003$) between this group and group of tumours with MN20 below first quartile (MN20 < 117.5%; arm a) was noticed. At least a 3-year local control was observed in 86% of patients in arm c representing MN20 over the median and only in about 42% in arm a (MN20 below first quartile). Significance was also achieved ($P = 0.04$) for both arms when overall survival was analysed.

Another analysis restricted to III B and IV stage (Figure 6) brings further clear evidence that lack of change, or only small increase of micronucleus frequency during the first course of radiotherapy, is an unfavourable prognosticator for tumour local control and total survival of patients. The 3-year local control was approximately 40% lower in the group with MN20 below 117.5% ($P < 0.007$) and total survival was lower by almost 30% ($P < 0.041$). Very similar relationships were obtained for the whole group II B–IV (data not illustrated), with significance at $P < 0.008$ and $P < 0.045$ for local control and total survival respectively. Although these data are not presented, it should be mentioned that increase of micronucleus frequency in vivo (MN20) was independent of patient's age ($r = -0.112$) and of haemoglobin level ($r = -0.123$). It is also seen from Table 2 that clinical parameters such as age and haemoglobin level had no influence on patient survival.

 *© 1999 Cancer Research Campaign*

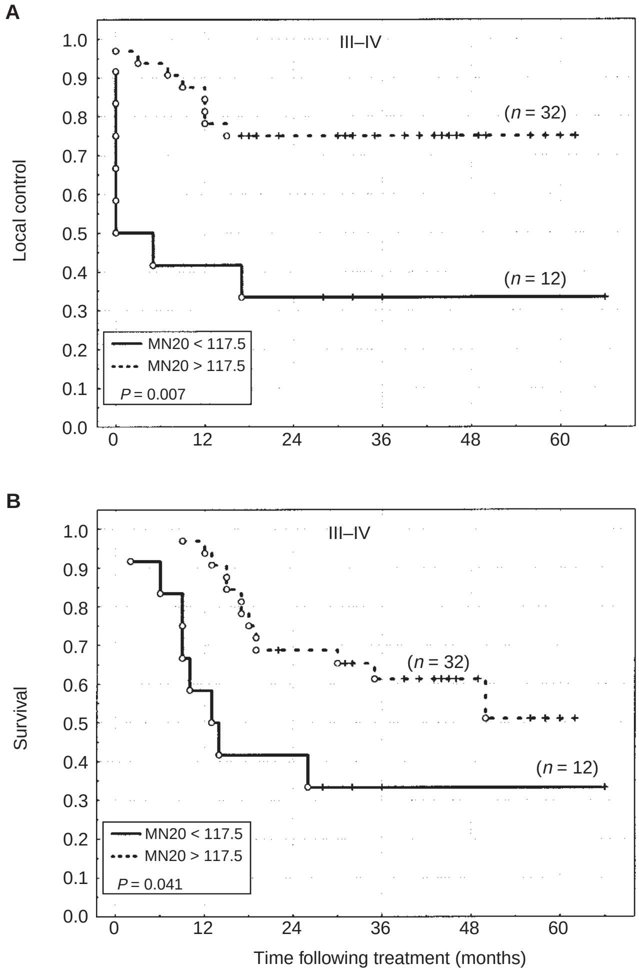

**Figure 6** Local control (**A**) and overall survival (**B**) probability vs increment of micronucleus frequency after 20 Gy in vivo for patients with stage III B–IV only. The upper arms in both panels represent MN20 over the first quartile and lower arms are for MN20 below first quartile

### DISCUSSION

The data presented by West et al (1993, 1997) clearly indicate that intrinsic radiosensitivity of cervical carcinomas measured as surviving fraction at 2 Gy in soft agar clonogenic assay can be used in predicting the outcome of patient treated by radiotherapy. However, methods based on cell survival are clinically not particularly applicable, mostly because of the long time (few weeks) needed to obtain final results. In our study we applied the cytokinesis-block micronucleus test as a possible alternative assay of tumour radiosensitivity in vitro. We also wanted to estimate simultaneously the frequency of micronuclei in tumour cells when radiation was administered to the tumour in situ. Usefulness of micronucleus assay for prediction of cellular radiosensitivity has not been definitively established yet. Reports about correlation between micronucleus frequency and reproductive cell death are controversial. A good correlation between clonogenic cell survival and micronucleus frequency in tumour cell lines was presented by several investigators (Wandl et al, 1989; Stap and Aten, 1990; Bakker et al, 1993). However, other studies brought contrasting results (Bush and McMillan, 1993; Slavotinek et al, 1993; Villa et al, 1994; Champion et al, 1995), indicating that lethal events measured as surviving fraction not always coincide with MN frequency. Unfortunately, micronucleus assay was always used to compare radiation sensitivity of established cell lines or primary culture of tumours of widely differing origin. The recent paper of Mariya et al (1997) based on the three murine tumour cell lines of the same origin but of different radiosensitivity evidenced strong correlation between frequency of micronuclei and clonogenic survival. Therefore, it seems that in order to evaluate the usefulness of micronucleus test as a predictive assay of radiosensitivity for clinical aims, only one tumour type should be used in a study. Our earlier report on a smaller group of patients (Widel et al, 1993) showed that both micronucleus frequency estimated in primary culture at clinically relevant doses 2–4 Gy, as well as micronucleus incidence induced by tumour irradiation in situ (10 × 2 Gy), widely differed among the tumours evaluated. The present paper comprises study results from 64 patients.

The prerequisite for successfully applying the cytokinesis-block micronucleus test to tumour cells isolated directly from biopsies was to obtain satisfactory cell outgrowth directly on plastic dishes. The success rate was 67%, which is roughly comparable to the 71% success rate for SF2 measured by West et al (1997) in soft agar colony assay. The radiation sensitivity at 2 Gy expressed as number of micronuclei per binucleated cell (MN/BNC) ranged from 0.08 to 0.416 (mean 0.224 ± 0.065). Number of MN/BNC was insignificantly lower ($P$ = 0.3) in stage II B tumours (0.199 ± 0.098) than in more advanced stages III–IV (0.231 ± 0.065) (data not presented). However, data were obtained for nine tumours in stage II B in comparison with 34 tumours in stage III–IV. Therefore, all data were used for correlation analysis between micronucleus frequency in vitro and clinical outcome. Although local control is slightly higher for tumours showing a higher number of MN/BNC, the difference is not significant (Figure 2A). There is no difference in overall survival of patients in relation to micronucleus frequency measured at 2 Gy in vitro. This is also confirmed by data presented in Table 2, where mean value of MN/BNC is even slightly higher for patients who died than that for those who were alive at the time of analysis.

Our results can not be directly compared with literature data. The only data evaluating correlation between response to radiotherapy of different human tumours with micronucleus frequency measured in primary culture as MN/BNC at 2 Gy indicate that such correlation exists (Shibamoto et al, 1994). However, this correlation concerns only early response to radiotherapy and is not precisely determined. It was also demonstrated (Shibamoto et al, 1998) that proliferative activity expressed as dividing fraction (DF) of tumour cells estimated on the basis of BNC plus multinucleated cells in CBMN assay highly correlated with relapse-free survival of patients with non-small-cell lung cancer. High DF was an unfavourable prognosticator. Although we have not performed similar comparison, the dividing fraction in cervix cancer seems to be low since we seldom observed multinucleated cells (0–3%) and average percentage of BNC was 15.84% in non-irradiated control. Low proliferative activity might be the reason that all damaged cells were not able to express micronuclei and thus estimated MN/BNC did not correlate with tumour responsiveness. Furthermore, in some samples the number of BNC counted per dish was even below 100. This is probably a common problem associated with CBMN assay on primary cultures of tumour cells, since in the study of Shibamoto et al (1998) only 50 BNC were scored per some dishes. In our study, correlation between binucleation yield and micronucleus frequency (MN/BNC at 2 Gy) is low

(*r* = 0.367), but it is in good agreement with the recent data presented by Johansen et al (1998) for human fibroblasts. Interestingly, these authors have not observed a correlation between either % BNC with MN (at 2 and 3.5 Gy) or clinical radiosensitivity assessed by excess risk of fibrosis.

In our study, binucleation percentage was comparable between the group of patients who died and the group who were alive at the time of analysis (Table 2), indicating that this parameter is also of minor importance for patient survival. The lack of correlation between cellular radiosensitivity estimated in CBMN assay and clinical outcome can probably be also ascribed partly to some selection of tumour cells during adaptation to artificial culture conditions. We observed that part of plated cells did not attach to the bottom of culture dishes and these cells were lost for the measurement of micronuclei. This problem is avoided in MN-assay after irradiation in vivo. Tumour biopsy after irradiation is mechanically disaggregated and cell suspension obtained contains all released cells. Thus it represents a larger population of tumour cells than the primary culture. The lack of correlation between spontaneous micronucleus frequency in vivo (MNSP) and in vitro (MN/BNC at 0 Gy), (Table 3) might support this supposition. Very low correlation was observed between MN/BNC at 2 Gy and MN20 in vivo (*r* = 0.324, Table 3). Although the index of radiation sensitivity in vitro does not correlate with clinical results, the increment of micronucleus frequency in vivo does. The micronucleus assay in vivo does not measure the intrinsic radiosensitivity separately, but overall radiation-induced cell damage that is influenced by environmental factors such as hypoxia, cellular proliferation, cell to cell contact and other host-related factors. Furthermore, MN20 reflects cytogenetic damage after fractionated irradiation, contrary to MN/BNC in vitro, estimated at single dose of 2 Gy. Although one can claim that MN20 measured in vivo is a rather crude parameter, it seems nevertheless useful for prediction of tumour response. A somewhat similar study was also performed on cervical cancer by Zölzer et al (1995). These authors observed that patients whose micronucleus frequency increased during radiotherapy together with diminution of S phase fraction had significantly better prognosis (5-year survival), although no single parameter evaluated could be judged statistically significant, probably because of insufficient number of patients included in this study.

Micronucleus increment after 20 Gy in our study seems to be a better predictor of local control than patient survival. When we analysed the causes of death in the group of patients with MN20 lower than median (<163.5% of pretreatment value), it appeared that 87% of patients died because of local recurrences and 13% died because of distant metastases. In the group of patients with MN20 >163.5% of pretreatment value, 40% of deaths were caused by recurrences and 60% by metastases and/or general health complications. In the group of patients with very low increment of MN20, below the first quartile (Figures 5 and 6), the cause of death was mainly a progression of disease (89%). These results mean that the higher increase in micronucleus frequency, the greater is the cell killing, and consequently greater is tumour response. Therefore, we can state that lack of change or only slight increase of MN frequency after 20 Gy in comparison with spontaneous MN frequency before radiotherapy strongly suggests that tumour probably will not be cured by radiotherapy alone and adjuvant treatment, e.g. chemotherapy should be considered after radiotherapy. In such a way, the increment of micronucleus frequency in vivo can offer a potentially valuable approach to predicting individual radiosensitivity and may also be of assistance in individualizing treatment strategy to improve cure rate in advanced stages of cervix cancer.

### ACKNOWLEDGEMENTS

This work has been conducted as the project No 2.5, Theme 3 S for 1997 within the Central Scientific Project of the Centre of Oncology.

### REFERENCES

Bakker PJM, Tukker LJ, Stap J, Veenhof CHN and Aten JA (1993) Micronuclei expression in tumours as a test for radiation sensitivity. *Radiother Oncol* **26**: 69–72

Bush C and McMillan TJ (1993) Micronucleus formation in human tumour cells: lack of correlation with radiosensitivity. *Br J Cancer* **67**: 102–106

Champion AR, Hanson JA, Court JB and Venables SE (1995) The micronucleus assay: an evaluation of its use in determining radiosensitivity in vitro. *Mutagenesis* **10**: 203–208

Davidson SE, West CML, Roberts SA, Hendry JH and Hunter RD (1990) Radiosensitivity testing of primary cervical carcinoma; evaluation of intra- and inter-tumour heterogeneity. *Radiother Oncol* **18**: 349–359

Fertil B and Malaise EP (1985) Intrinsic radiosensitivity of human cell lines is correlated with radioresponsiveness of human tumors. Analysis of 101 published survival curves. *Int J Radiat Oncol Biol Phys* **11**: 1699–1707

Girynsky T, Lubin R, Pignon JP, Chavaudra N, Gazean J, Dubray B, Cosset JM, Socie G and Fertil B (1992) Predictive value of in vitro radiosensitivity parameters in head and neck cancers and cervical carcinomas: preliminary correlations with local control and overall survival. *Int J Radiat Oncol Biol Phys* **25**: 3–7

Heddle JA and Carrano AV (1977) The DNA content in micronuclei induced in mouse bone marrow by gamma irradiation: evidence that micronuclei arise from acentric chromosomal fragments. *Mutat Res* **44**: 63–69

Johansen J, Streffer C, Fuhrmann C, Bentzen SM, Stausbøl-Grøn B, Overgaard M and Overgaard J (1998) Radiosensitivity of normal fibroblasts from breast cancer patients assessed by the micronucleus and colony assays. *Int J Radiat Biol* **73**: 671–678

Mariya Y, Streffer C, Fuhrmann C and Wojcik A (1997) Correlation of radiation-induced micronucleus frequency with clonogenic survival in cells of one diploid and two tetraploid murine tumor cell lines of the same origin. *Radiat Res* **147**: 29–34

Midander J and Revesz L (1980) The frequency of micronuclei as a measure of cell survival in irradiated cell populations. *Int J Radiat Biol* **38**: 237–242

Schwartz JL, Mustafi R, Beckett MA, Czyżewski EA, Farhangi E, Grdina DJ, Rotmensch R and Weichselbaum RR (1991) Radiation-induced DNA double-strand break frequencies in human squamous cell lines of different radiation sensitivities. *Int J Radiat Biol* **59**: 1341–1352

Schwartz JL, Mustafi R, Beckett MA and Weichselbaum RR (1996) DNA double-strand break rejoining rates, inherent radiation sensitivity and human tumour response to radiotherapy. *Br J Cancer* **74**: 37–42

Shibamoto Y, Streffer C, Fuhrmann C and Budach V (1991) Tumor radiosensitivity prediction by the cytokinesis-block micronucleus assay. *Radiat Res* **128**: 293–300

Shibamoto Y, Shibata T, Miyatake S, Oda Y, Manabe T, Ohshio G, Yagi K, Streffer C, Takahashi M and Abe M (1994) Assessment of the proliferative activity of human tumours using the cytokinesis-block micronucleus assay. *Br J Cancer* **70**: 67–71

Shibamoto Y, Ike O, Shibata T, Takahashi M, Abe M and Streffer C (1998) The cytokinesis-block micronucleus assay for the prediction of proliferative activity and radiosensitivity of human tumors. In: *Progress in Radio-Oncology VI*, Kogelnik HD and Sedlmayer F (eds), pp. 509–515. Munduzzi Editore: Bologna, Italy

Slavotinek A, McMillan TJ and Steel CM (1993) A comparison of micronucleus frequency and radiation survival in lymphoblastoid cell lines. *Mutagenesis* **8**: 569–575

Stap J and Aten JA (1990) Comparison of radiation sensitivity for three cell lines as measured by the cloning assay and the micro-nucleus test. *Strahlenther Onkol* **166**: 761–763

Villa R, Zaffaroni N, Gornati D, Costa A and Silvestrini R (1994) Lack of correlation between micronucleus formation and radiosensitivity in established and primary cultures of human tumours. *Br J Cancer* **70**: 1112–1117

 *© 1999 Cancer Research Campaign*

Wandl EO, Ono K, Kain R, Herbsthofer T, Heinert G and Hobarth K (1989) Linear correlation between surviving fraction and the micronucleus frequency. *Int J Radiat Biol* **56**: 771–775

Weissenborn U and Streffer C (1991) Micronuclei with kinetochores in human melanoma cells and rectal carcinomas. *Int J Radiat Biol* **59**: 373–383

West CML and Hendry JH (1992) Intrinsic radiosensitivity as a predictor of patient response to radiotherapy. *Br J Radiol* **24**: 146–152

West CML, Davidson SE, Roberts SA and Hunter RD (1993) Intrinsic radiosensitivity and prediction of patient response to radiotherapy for carcinoma of the cervix. *Br J Cancer* **68**: 819–823

West CML, Davidson SE, Roberts SA and Hunter RD (1997) The independence of intrinsic radiosensitivity as a prognostic factor for patient response to radiotherapy of carcinoma of the cervix. *Br J Cancer* **76**: 1184–1190

Whitaker SJ, Ung YC and McMillan TJ (1995) DNA double-strand break induction and rejoining as determinants of human tumour cell radiosensitivity. A pulsed-field gel electrophoresis study. *Int J Radiat Biol* **67**: 7–18

Widel M, Dobrut M, Maka B, Lubecka B, Pluciennik A (1997) The radiosensitivity of human malignant melanomas evaluated by cytokinesis-block micronucleus assay. *Neoplasma* **44**: 109–116

Widel M, Konopacka M, Jedrus S and Owczarek S (1993) Evaluation of the human cervical carcinoma cells radiosensitivity by the micronucleus assay. *Abstracts from Annual Meeting of ESRB*, Stockholm, Sweden

Zamboglou N, Streffer C, Fritzmeir CU, Bojar H, Karstens JH, Schnabel T, Kolotas C and Schmitt G (1992) Klinische Prognose Faktoren der Radioresistenz by Kopf-Hals-Karzinomen. *Dtsch Z Mund Kiefer Gesichts Chir* **16**: 82–85

Zölzer F, Alberti W, Pelzer T, Lamberti G, Hulskamp FH and Streffer C (1995) Changes in S phase fraction and micronucleus frequency as prognostic factor in radiotherapy of cervical carcinoma. *Radiother Oncol* **36**: 128–132

© 1999 Cancer Research Campaign 
